# Potential normal tissue sparing in vulvar cancer using intensity modulated proton therapy versus volumetric modulated arc therapy

**DOI:** 10.1016/j.phro.2026.101004

**Published:** 2026-05-27

**Authors:** Anna C. Prins, Raymond de Boer, András G. Zolnay, Remi A. Nout, Mischa S. Hoogeman, Kelvin Ng Wei Siang

**Affiliations:** a Holland Proton Therapy Center, Delft, the Netherlands; b Department of Radiotherapy, Erasmus MC Cancer Institute, University Medical Center Rotterdam, Rotterdam, the Netherlands

**Keywords:** Vulvar cancer, Intensity modulated proton therapy, Volumetric modulated arc therapy, Robust optimization, Normal tissue complication probability

## Abstract

**Background and purpose:**

Photon radiotherapy, typically volumetric modulated arc therapy (VMAT), is the standard for adjuvant or definitive treatment of vulvar cancer. Here, we explore the potential of intensity modulated proton therapy (IMPT) to further reduce radiation side effects.

**Materials and methods:**

Thirty patients treated with VMAT (59.4–64.5 Gy(RBE, relative biological effectiveness)) to boost clinical target volume (CTV), 45–49.5 Gy(RBE) to the elective CTV, in 27–33 fractions) were retrospectively planned with IMPT, using four and six beams with simultaneous integrated boost (SIB) or sequential (SEQ) techniques. Target coverage and organs of interest (OOI) dose constraints were evaluated for bladder, bowel bag, anorectum, femoral heads, pelvic bone and skin. Normal tissue complication probabilities (NTCPs) were calculated for eight endpoints. Skin dose–surface distributions and NTCP–target volume trends were analyzed.

**Results:**

The IMPT plans achieved robust target coverage similar to VMAT while significantly reducing OOI doses (p < 0.001; e.g., lower pelvic bone marrow V_5Gy__(RBE)_, VMAT: 100 [100–100]%, IMPT: 62.9 [31.7–97.8]%), and the NTCPs (e.g., hematologic side effects grade ≥ 3, VMAT: 19.4 [4.6–50.2]%, IMPT: 2.3 [0.4–9.5]%). Despite similar grade 3 dermatitis risk (VMAT:79.6 [14.0–99.2]%, IMPT: 76.3 [17.9–96.6]%, p < 0.001), localized high-doses were observed at the lower abdominal/inguinal skin. The NTCPs increased with increasing target volume, less so for IMPT. Six-beam and SIB plans improved robustness and OOI sparing over four-beam and SEQ.

**Conclusions:**

Besides target coverage, robustly optimized IMPT achieved substantial OOI dose and NTCP reductions versus VMAT in vulvar cancer. Clinical implementation requires attention to high-dose skin regions.

## Highlights

• Proton therapy achieved target coverage comparable to photon therapy in vulvar cancer.
• Large organ at risk dose reductions: e.g. 10 Gy, bladder D35 in protons vs photons.
• Modeled toxicity risks decreased too: hematologic toxicity ≥ 3 drops, 19.4% to 2.3%.
• Proton therapy may increase localized skin dose in lower abdominal/inguinal regions.
• Six-beams were generally better than four-beams: femoral head D35 drops by ∼ 1.9 Gy.

## Graphical abstract

**Figure:**
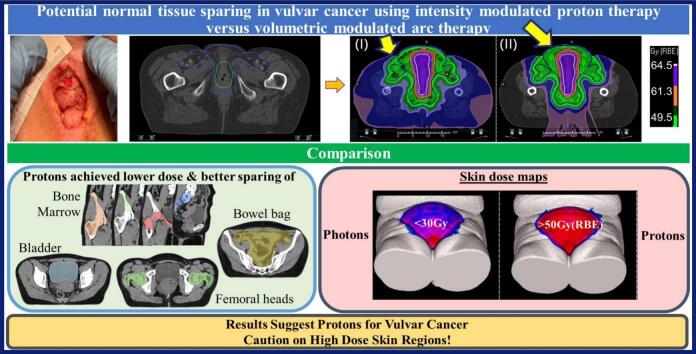

## 1 Introduction

Vulvar cancer (mainly squamous cell carcinoma) is the 21st most common cancer in women worldwide, with 537 new cases and 166 deaths in the Netherlands in 2022 [1], [2]. Treatment depends on tumor stage and location: while early-stage disease is treated with a radical local excision and sentinel node procedure, adjuvant (chemo)radiotherapy is recommended for positive margins, high risk of recurrence, multiple lymph node metastases, or extracapsular extension. Chemoradiotherapy is the primary treatment for advanced disease, with surgery for no complete response [3].

Over 25% of patients receiving photon intensity modulated radiation therapy (IMRT) for vulvar cancer experience severe (grade 3) acute side effects, including dermatological, genitourinary (GU), gastrointestinal (GI) and hematologic complications [4]. Long-term effects include fibrosis, edema, vaginal dryness, pain, stenosis, and reduced bladder and bowel function, which can significantly impact sexual function and quality of life [5].

No clinical trials or dose impact studies have yet explored proton therapy for vulvar cancer. However, its benefits [6] have been demonstrated in other gynecologic (e.g., cervical and endometrial) cancers. Intensity modulated proton therapy (IMPT) improves organs of interest (OOI) sparing compared to IMRT or volumetric modulated arc therapy (VMAT), and reduces potential side effects [7], [8], [9], [10]. Similarly, IMPT could reduce radiotherapy side effects in vulvar cancer. However, skin-related side effects may increase [11] due to its lower skin-sparing effect compared to photons [12].

The aim of this study was to fully investigate the potential of proton therapy for vulvar cancer, and provide guidance for clinical introduction.

## 2 Materials and methods

### 2.1 Patient characteristics

All patients were treated at Erasmus Medical Center Rotterdam. The study was approved by the Medical Ethics Committee of Erasmus MC, reference MEC-2025–0208. The inclusion criteria are given in Supplemental Material A.

The cohort included 30 patients with locally advanced or recurrent disease. Patient characteristics are summarized in Table S3.A.1. Patients receiving primary chemoradiotherapy were treated with a sequential boost (SEQ) scheme from 2020 to 2022 and a simultaneous integrated boost (SIB) scheme from 2023 onward. Adjuvant chemoradiotherapy was delivered exclusively with a SIB schedule. Fractionation schemes and dose prescription details for all groups are provided in Table 1. All doses are reported in Gray relative biological effectiveness (Gy(RBE)^1^).

**Table 1:** Fractionation scheme details for the study population.

**Treatment type**	**Delivery**	**Boost CTV absolute dose (Gy(RBE))**	**Elective CTV absolute dose (Gy(RBE))**	**Boost fractionation scheme**	**Elective fractionation scheme**
Primary	SIB	64.5 (vulva), 57 (nodal)	49.5	30 x 2.15 Gy(RBE) (vulva), 30 x 1.9 Gy(RBE) (nodal)	30 x 1.65 Gy(RBE)
Primary	SEQ	59.4 (vulva)	45	25 x 1.8 Gy(RBE)	8 x 1.8 Gy(RBE)
Adjuvant	SIB	59.4 (vulva), 55.35 (nodal)	48.6	27 x 2.20 Gy(RBE) (vulva), 27 x 2.05 Gy(RBE) (nodal)	27 x 1.62 Gy(RBE)

SEQ = sequential boost, SIB = simultaneous integrated boost.

### 2.2 Target and normal tissue delineation

A planning computed tomography (CT) scan was performed for all patients in a head-first-supine position. Gross tumor volume (GTV), boost and elective clinical target volumes were delineated by radiation oncologists, following Radiation Therapy Oncology Group (RTOG) guidelines [15]. For the OOIs, the bladder, femoral heads, anorectum, bowel bag (using the MIM Contour *ProtégéAI +* model; MIM Software Inc, USA [16]), bone marrow [17], [18] and skin (3 mm shell from the patient surface) were delineated. All delineation details are provided in Supplemental Material A, with target volume dimensions in Table S3.A.2.

### 2.3 Treatment planning

Clinically approved VMAT plans were used, and treatment planning was done using Monaco (Elekta AB, Sweden). To account for uncertainties in patient setup and VMAT delivery, the clinical target volume (CTV) was expanded isotropically into a planning target volume (PTV) (8–10 mm for vulvar elective CTV, 7 mm for lymph nodes, and 5 mm for boost CTV) based on institutional protocols. Radiotherapy was delivered with 6 or 10 MV photon beams using two arcs (185–360° gantry angles with 5° collimator). Dose calculations were performed with the X-ray Voxel Monte Carlo or Monte Carlo Photon algorithm, with a statistical uncertainty of 0.75%–5% (Monaco, Elekta AB, Sweden).

Robust IMPT planning was performed retrospectively using RayStation (Raysearch Laboratories AB, Sweden), creating two plans per patient with four (30°, 160°, 200°, 330°) and six (30°, 90°, 160°, 200°, 270°, 330°) pencil beam scanning beams. Since VMAT’s CTV-PTV concept does not account for proton therapy range uncertainties, composite minimax robust optimization [19] for IMPT was used, incorporating uncertainty information to ensure adequate target coverage [19], [20]. Using 5 mm setup and 3% range uncertainties, 28 scenarios were simulated [21]. To match CTV-PTV margins from VMAT, the vulvar elective CTV was expanded by 3–5 mm, and the inguinal CTV by 2 mm, following institutional guidelines. Prescription dose and fractionation matched the VMAT plans, and optimization focused on robust target coverage while minimizing OOI doses. Dose calculations were performed using Monte Carlo [22], with a statistical uncertainty of 1%.

### 2.4 Target coverage and OOI constraints evaluation

Target coverage was assessed using D_98__%_ and D_2__%_ [23]. For VMAT, the PTV was evaluated on the clinical nominal plans (boost PTV D_98__%_ ≥ 95% and D_2__%_ < 107%, elective PTV D_98__%_ ≥ 95%). For IMPT, similar criteria were followed for the boost (D_98__%_ ≥ 95%, D_2__%_ < 107%) and elective CTVs (D_98__%_ ≥ 95%), using voxel-wise minimum and maximum dose distributions. Consistency between VMAT nominal PTV–based metrics and IMPT voxel–wise worst–case CTV–based metrics has been previously established [24].

All OOIs were evaluated based on dose constraints listed in Table S3.B.1, based on RTOG 0529 [25], RTOG 0418 [26], Gynecologic Oncology Group 279 [27] and other studies [28], [29]. Clinical acceptability of IMPT plans was also evaluated by a radiation oncologist with 18 years of experience in treating vulva cancers.

### 2.5 Additional analyses and normal tissue complication probabilities

A comparison between SIB and SEQ was conducted to assess the effects on target coverage and OOI doses. Details are provided in Supplementary Material B.

For exploratory analysis, eight normal tissue complication probability (NTCP) models from different disease sites were applied to estimate radiation-induced adverse events across the OOIs defined [30], [31], [32], [33], [34], [35], [36], [37], [38], [39]. Input parameters included equivalent uniform dose (EUD) and dose-volume histograms (DVH). For the skin model, the relative skin surface receiving > 20 Gy(RBE) (S_20Gy(RBE)_) was used as a predictor for skin side effects [37], with relative dose-surface histograms (DSHs) as input [38], [39], [40]. All endpoints are in Supplemental Material A. The NTCP functions and variables are in Supplementary Material 3.C.

Three-dimensional (3D) spatial dose-surface maps (low (5–25 Gy(RBE)) and high-dose (≥ 25 Gy(RBE)) regions) were generated and visualized for VMAT and IMPT plans using an in-house Python script. Details are provided in Supplemental Material A.

The effect of tumor size on NTCP was further explored by examining the correlation between NTCP and target volumes. Detailed methodology and results, including Spearman’s rank correlation *ρ* [41] and linear regression analysis, are provided in Supplementary Material B.

Differences between groups were tested using the Wilcoxon-Mann-Whitney test due to the sample size of thirty. A 5% significance threshold (p = 0.05) was applied. All analyses were performed with Python 3.11.9 (Table 2).

**Table 2:** Comparison of target coverage metrics between VMAT, IMPT-4B and IMPT-6B. VMAT treatment plans were evaluated based on the PTV of the nominal plans, IMPT plans on the CTV on the voxel-wise minimum and maximum dose distributions (Voxmin/max). Values are median (%) [range], expressed as percentage of the prescribed dose for a particular target. The p-values compare VMAT with IMPT-4B, VMAT with IMPT-6B and IMPT-4B with IMPT-6B, respectively. Significant p-values are indicated in bold.

**Target**	**Constraint**	**VMAT** (PTV nominal) Median [range] (%)	**IMPT 4 Beams** (CTV Voxmin/max) Median [range] (%)	**IMPT 6 Beams** (CTV Voxmin/max) Median [range] (%)	*p***-value** (VMAT vs IMPT-4B)	*p***-value** (VMAT vs IMPT-6B)	*p***-value** (IMPT-4B vs IMPT-6B)
Boost	D_98__%_ ≥ 95%	96.5 [94.6–98.6]	97.4 [95.0–98.9]	97.6 [95.3–98.1]	**0.014**	*<***0.001**	*<***0.001**
	D_2__%_ ≤ 107%	103.1 [101.7–104.8]	105.6 [104.0–107.7]	105.3 [100.3–106.7]	*<***0.001**	*<***0.001**	*<***0.001**
Elective	D_98__%_ ≥ 95%	97.1 [95.1–104.6]	97.4 [96.0–98.9]	97.5 [95.7–100.3]	0.839	0.289	*<***0.001**

## 3 Results

**Fig. 1:**
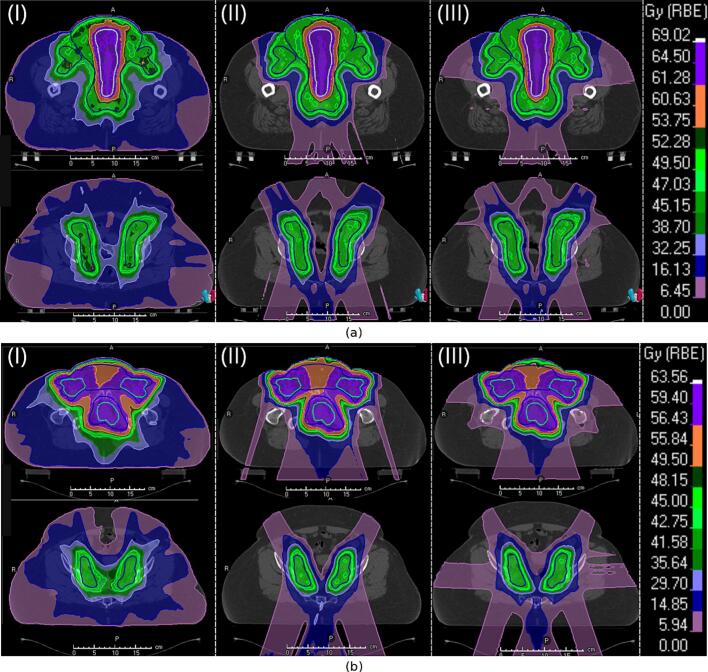
Cross-sectional dose distributions at the level of the boost and elective CTVs for two patients with a SIB (a) and SEQ (b) fractionation schedule. The CTV delineations are shown in dark blue (elective CTV) and light green (boost CTV). I = VMAT; II = IMPT-4B; III = IMPT-6B. (For interpretation of the references to colour in this figure legend, the reader is referred to the web version of this article.)

Fig. 1a shows the dose distributions for a patient planned with a SIB fractionation scheme, and Fig. 1b with a SEQ scheme, each for (I) VMAT, (II) four-beam IMPT (IMPT-4B), and (III) six-beam IMPT (IMPT-6B). Visual inspection showed a steeper dose fall-off in SIB plans compared to SEQ plans. The VMAT plans showed a larger low-dose bath compared with IMPT.

All IMPT plans achieved adequate target coverage. Compared to the nominal VMAT plans, the voxel-wise worst case IMPT plans showed significantly better PTV coverage for the boost volume receiving ≥ 95% of the dose (p < 0.05), but no significant difference for the elective CTVs. The high-dose constraint (D_2__%_ < 107%) was significantly lower for VMAT than both IMPT plans (p < 0.001). All differences between IMPT-4B and IMPT-6B were statistically significant (p *<* 0.001), although they were small (0.1–0.3% median differences).

**Fig. 2:**
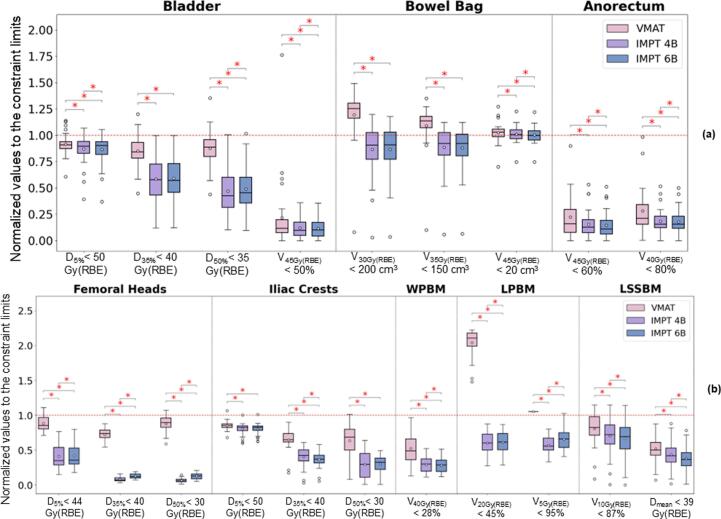
Box-and-whisker plots evaluating OOI dose constraints listed in Table S3.B.1. The y-axis shows values normalized to the constraint limits (red line = 1) for VMAT, IMPT-4B, and IMPT-6B plans. Black line = median, white dot = mean, box edges = interquartile range (Q1–Q3), whiskers = range, crosses = individual outliers. Red asterisks indicate statistically significant differences (p < 0.05). (For interpretation of the references to colour in this figure legend, the reader is referred to the web version of this article.)

Fig. 2 presents the evaluation of OOI doses against dose constraints for VMAT, IMPT-4B, and IMPT-6B. Full data are in Table S3.E.1. Both IMPT plans achieved significantly lower median values for all constraints than VMAT (p < 0.05).

Femoral heads and lower pelvic bone marrow (LPBM) showed the largest dose reductions. The LPBM V_5Gy__(RBE)_ decreased from 100 [100–100]% with VMAT to 52.8 [31.7–76.5]% with IMPT-4B and 62.9 [39.1–97.8]% with IMPT-6B, and V_20Gy__(RBE)_ decreased from 94.8 [66.6–100]% to 27.2 [12.5–39.5]% and 27.6 [12.9–39.1]%, respectively (p < 0.001). Femoral head D_5__%_, D_35__%_, and D_50__%_ decreased from 37.5 [31.4–48.8], 29.5 [21.8–35.3], and 20.5 [2.5–30.3] Gy(RBE) with VMAT to 15.5 [6.8–33.7], 2.9 [1.4–6.4], and 1.9 [0.4–4.3] Gy(RBE) for IMPT-4B, and 15.7 [8.1–35.1], 4.8 [3.0–7.7], and 3.8 [1.7–6.3] Gy(RBE) for IMPT-6B (p < 0.001).

Notable reductions were also seen in the bladder and bowel bag. Bladder D_35__%_ decreased from 33.6 [17.9–48.1] Gy(RBE) with VMAT to 23.1 [4.8–39.9] Gy(RBE) with IMPT-4B and 22.8 [4.9–39.9] Gy(RBE) with IMPT-6B (p < 0.001), and D_50__%_ from 31.0 [15.3–47.3] Gy(RBE) to 14.9 [3.6–35.2] and 15.8 [3.3–35.5] Gy(RBE), respectively (p < 0.001). Bowel bag V_30Gy__(RBE)_ decreased from 251 [16.7–299] cm^3^ with VMAT to 181 [6.0–239] cm^3^ with IMPT-4B and 182 [67.3–236] cm^3^ with IMPT-6B (p < 0.001).

For the femoral heads, LPBM, and most bladder constraints IMPT-4B achieved lower doses, while IMPT-6B performed better for iliac crest D_35__%_, bowel bag V_45Gy__(RBE)_, and all rectum, WPBM, and LSSBM constraints. However, differences between IMPT-4B and IMPT-6B were generally small (< 2 Gy(RBE)).

Results comparing SIB and SEQ for target coverage and doses to the OOIs are provided in Supplementary Material B and Supplementary Material 3.D and 3.E.

**Fig. 3:**
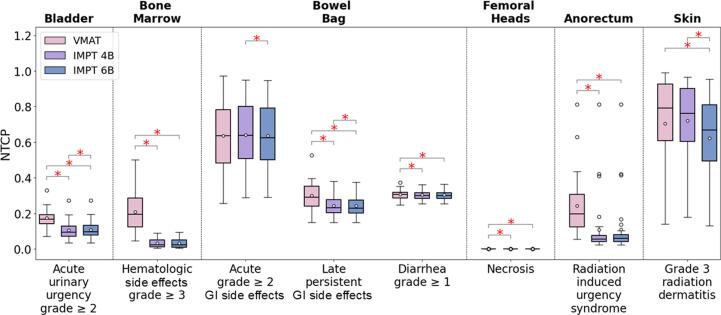
Box and whisker plots to compare the estimated Normal Tissue Complication Probability (NTCP) on the VMAT, IMPT-4B and IMPT-6B plans for the bladder, bone marrow, bowel bag, femoral heads, rectum and skin, using the models described in Supplementary Material 3.C. In each plot, the black line, white dot, edges of the box, whiskers, and crosses represent the median, mean, interquartile range (Q1-Q3), range and individual outliers, respectively. Red asterisks indicate statistically significant comparisons: * p < 0.05. (For interpretation of the references to colour in this figure legend, the reader is referred to the web version of this article.)

Fig. 3 shows exploratory results of NTCP for eight side effect endpoints across VMAT, IMPT-4B, and IMPT-6B plans for the defined OOIs, with the corresponding data provided in Table S3.F.1. The IMPT plans showed significantly lower NTCPs than VMAT for nearly all endpoints. Bladder risk for acute urinary urgency grade ≥ 2 decreased from 16.8 [7.2–33.0]% with VMAT to 9.4 [3.4–27.3]% and 9.7 [4.3–27.3]% for IMPT-4B and IMPT-6B, respectively (p < 0.001). Hematologic side effects risk ≥ 3 decreased from 19.4 [4.6–50.2]% with VMAT to 2.3 [0.4–8.9]% and 2.2 [0.4–9.5]% with IMPT-4B and IMPT-6B (p < 0.001), respectively.

Proton plans also decreased the risk of rectal radiation-induced urgency syndrome (VMAT: 19.8 [5.6–81.4]% vs IMPT-4B: 5.6 [2.2–81.4]%, IMPT-6B: 6.0 [2.2–81.4] %, p < 0.001). For bowel bag endpoints, differences between VMAT and IMPT were insignificant for acute GI side effects (p = 0.626, 0.440). However, late persistent GI side effects were lower with IMPT (VMAT: 29.2 [14.8–52.7]%, IMPT-4B: 23.1 [15.0–37.9]%, IMPT-6B: 23.0 [15.0–37.6]%, p < 0.001), as was diarrhea grade ≥ 1. Femoral head necrosis NTCPs were low for all plans, but IMPT significantly outperformed VMAT (p < 0.001). No significant difference in grade 3 radiation dermatitis was found between VMAT and IMPT-4B (p = 0.626). However, IMPT-6B showed significantly lower NTCP (66.9 [13.1–95.4]%) than both VMAT (79.6 [14.0–99.2]%) and IMPT-4B (76.3 [17.9–96.6]%, p < 0.001), indicating potential less skin side effects.

**Fig. 4:**
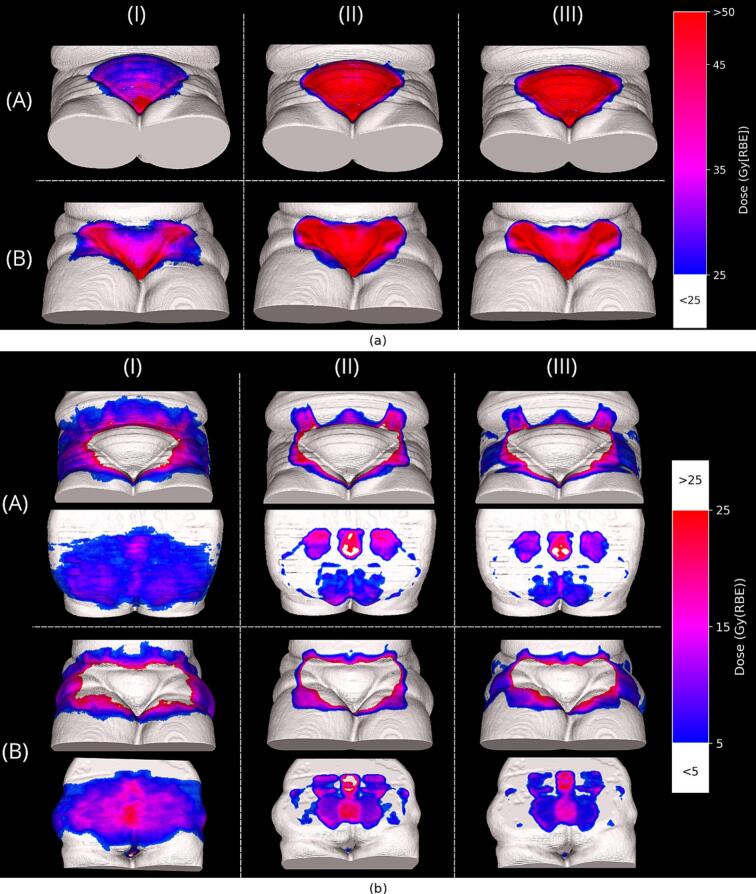
The 3D skin dose-surface maps for two patients showing 25–50 Gy(RBE) (a) and 5–25 Gy(RBE) (b). I = VMAT; II = IMPT-4B; III = IMPT-6B. Fig. 4b shows frontside and backside views. Patient 1: SIB, 30 fractions (boost 64.5 Gy(RBE), elective 49.5 Gy(RBE)); Patient 2: SEQ, 33 fractions (boost 59.4 Gy(RBE), elective 45 Gy(RBE)).

Fig. 4 shows 3D dose-surface maps of the skin for two patients for high-dose ≥ 25 Gy(RBE) (Fig. 4a) and low-dose 5–25 Gy(RBE) (Fig. 4b). For ≥ 25 Gy(RBE), IMPT plans showed a higher dose, particularly above 50 Gy(RBE) in the lower abdominal and inguinal skin areas with deep skin folds. Compared with IMPT-4B, IMPT-6B showed lower high-dose exposure in these regions. For 5–25 Gy(RBE), VMAT showed a more diffuse dose distribution over a larger surface of skin, while IMPT had more patchy, much smaller low-dose regions.

The correlation between NTCP and target volumes for late persistent GI side effects and radiation dermatitis, along with data for other endpoints, are provided in Supplementary Material B and Supplementary Material 3.G.

## 4 Discussion

In this IMPT versus VMAT treatment planning study, robust and clinically acceptable target coverage was achieved with IMPT while reducing doses to multiple organs at risk and lowering NTCPs across several side effect endpoints compared to VMAT. Dermatitis risk was comparable to VMAT, however, dose–surface maps showed potential increased localized skin dose in lower abdominal/inguinal regions.

All IMPT plans were accepted by our radiation oncologist. Robust target coverage was achieved in all IMPT plans. Six-beam IMPT was slightly more robust than four beams. For the boost target, median D_98__%_ and D_2__%_ values were statistically significantly higher than VMAT, while median D_98__%_ for the elective target was comparable to VMAT. Both four and six beams therefore provided clinically acceptable target coverage for vulvar cancer.

The IMPT plans reduced doses to all OOIs and most NTCP predictions compared to VMAT, especially for bone marrow and femoral heads, corresponding to lower risks of radiation-induced fractures and necrosis [42], [43]. Reduced doses to the iliac crests, femoral heads, whole pelvic bone marrow (WPBM), LPBM, and lumbosacral spine bone marrow (LSSBM) may also lower risks of hematologic side effects, fractures, and bone mineral density loss [18], [28]. The NTCP models predicted up to 88% lower hematologic side effects and no femoral head events, achieved without bone marrow sparing objectives. These findings support previous studies showing IMPT can reduce bone marrow dose and hematologic NTCP without compromising other OOIs [10], [44]. While VMAT can implement bone marrow sparing, excessive sparing may increase doses to other OOIs [21], highlighting IMPT’s advantage in lowering modeled hematologic risk.

Statistically significant reductions in OOI doses were also observed for the bladder, bowel bag, and anorectum, potentially reducing gastrointestinal, genitourinary, and rectal side effects [25], [26]. For all but one endpoint, NTCP was reduced. While RTOG 0529 bowel bag constraints (V_30Gy__(RBE)_ < 200 cm^3^, V_35Gy__(RBE)_ < 150 cm^3^and V_45Gy__(RBE)_ < 20 cm^3^) were not fully met in some patients, these constraints were defined for individual bowel loops, not the whole bowel bag [25]: their application may be conservative. At our center, these RTOG constraints are not used. Still, IMPT's reduction in bowel bag dose and NTCP indicates its potential to reduce GI side effects compared to VMAT. Note that OOI dose reductions with IMPT could be even greater if the boost CTV coverage were scaled to match the boost D_98__%_ prescription.

Patients with vulvar cancer treated with photons often develop grade 2–3 dermatitis [4], [45]. Unlike photons, protons lack skin-sparing effects, which can increase side effects. The NTCP for grade 3 dermatitis was similar for VMAT and IMPT-4B, but IMPT-6B showed lower risk, suggesting that six beams may reduce skin damage. Dose calculation uncertainties near the surface have negligible impact on the NTCP model [40]. Nevertheless, dose-surface maps revealed IMPT produced more high-dose regions (> 50 Gy(RBE)) at the lower abdomen and inguinal skin, potentially increasing localized dermatitis. Skin side effects remain an important consideration for IMPT for vulvar cancer.

Both SIB and SEQ provided robust target coverage and adequate OOI sparing. However, SIB resulted in steeper dose gradients and larger reductions in OOI doses (Supplementary Material B). Therefore, SIB is preferable for IMPT in vulvar cancer, consistent with studies comparing between SIB and SEQ [46], [47]. The IMPT plans maintained its advantage over VMAT regardless of the boost method.

A slight positive correlation was observed between elective CTV volume and late persistent GI side effects, and between GTV/boost CTV volume and grade 3 radiation dermatitis risk (Supplementary Material B), but weak correlations for other endpoints (Supplementary Material 3.G). However, IMPT showed a smaller or similar NTCP increase with larger target volumes compared to VMAT, indicating that IMPT maintains its dose advantage in larger tumors.

Although minimal, IMPT-6B showed greater robustness than IMPT-4B, especially in the SIB plans. Both IMPT configurations improved OOI sparing compared to VMAT, with IMPT-4B slightly better sparing the femoral heads and lower pelvic bone marrow, while IMPT-6B was slightly better for the iliac crests and other bone marrow constraints. More conservative high-dose skin regions and a lower NTCP for grade 3 radiation dermatitis were also shown in IMPT-6B, suggesting it may reduce skin side effects with minimal dose impact on other OOIs. However, the choice for beam setup is influenced by other clinical factors such as patient anatomy, optimization complexity, and treatment time.

The NTCP models used provide insight into potential clinical relevance but have limitations. No vulvar cancer specific models exist. Most models are based on photon therapy from other pelvic sites, with differences in patient populations, fractionation, tumor location, dose distributions, and modalities; only the skin model [37] is derived from proton therapy data, though it lacks broader validation. Most rely on DVH or DSH parameters (e.g., EUD; V_30Gy__(RBE)_; S_20Gy__(RBE)_), assuming uniform organ responses and ignoring patient-specific factors. Despite these limitations, observed differences between VMAT and IMPT are expected to hold, as all plans were evaluated with the same methodology.

Besides NTCP, 30 patients are modest and heterogeneous in tumor stage, chemotherapy, and fractionation schemes, though adequate for a within-patient comparison between VMAT and IMPT. Larger, more homogeneous datasets would improve precision and statistical power, but the main conclusions are expected to hold. Additionally, all evaluations were based on planned, and not delivered doses. Dose accumulation using daily scans (e.g., cone-beam CT, CBCT)) could be considered [48] to assess the impact of anatomical and setup variations on plan quality.

Following the Dutch model-based approach, patients are often referred to proton therapy if NTCP models predict significant side effect reductions compared to photons [49]. Treatment decisions weigh also dosimetry, clinical experience, and patient factors. While no formal model-based indication exists for vulvar cancer, our results showing > 10% OOI dose and NTCP reductions suggest it could be considered, even without full model-based decision making. Potential skin side effects with IMPT may limit its benefit and preclude referral. At our clinic, skin reactions are closely monitored and managed early using topical agents such as cetomacrogol, calendula, and lanette.

In this work, we used a robustness envelope in our treatment planning, which included a margin to account for anticipated anatomical shifts. However, this may not fully capture all errors during treatment. Anatomical changes such as weight loss, evolving edema, skin fold changes, and variable bowel gas, may lead to shifts in target volumes and OOI positions. These may also affect dosimetry and OOI sparing during treatment; however, adaptive replanning could offer a potential solution.

In our clinic, daily CBCT verifies position, focusing on bony anatomy and target volumes. Significant anatomical changes or tumor growth/shrinkage prompt a control CT after discussion with the radiation oncologist and medical physicist. If CTV coverage is insufficient, the adaptive workflow is triggered using the control CT as the new planning CT; transient changes may delay adaptation by one or two fractions. This approach can preserve IMPT’s robustness and dose-volume benefits, though further research is needed to define precise replanning criteria and trigger protocols.

## CRediT authorship contribution statement

**Anna C. Prins:** Writing – review & editing, Writing – original draft, Visualization, Methodology, Investigation, Formal analysis, Data curation, Conceptualization. **Raymond de Boer:** Writing – review & editing, Writing – original draft, Validation, Investigation, Data curation, Conceptualization. **András G. Zolnay:** Visualization, Software, Resources, Methodology, Data curation. **Remi A. Nout:** Writing – review & editing, Writing – original draft, Methodology, Formal analysis, Conceptualization. **Mischa S. Hoogeman:** Writing – review & editing, Writing – original draft, Visualization, Supervision, Resources, Formal analysis, Conceptualization. **Kelvin Ng Wei Siang:** Writing – review & editing, Writing – original draft, Visualization, Validation, Supervision, Software, Project administration, Methodology, Investigation, Formal analysis, Data curation, Conceptualization.

## Declaration of competing interest

The authors declare that they have no known competing financial interests or personal relationships that could have appeared to influence the work reported in this paper.

## Appendix A Supplementary data

Supplementary Data 1 Supplementary Information including additional methodological details, analyses, and supporting results.

## Footnotes

1 For VMAT, an RBE of 1.0 was applied, while for IMPT an RBE of 1.1 was used, in line with clinical practice to account for the higher biological effectiveness of protons [13], [14].
