# Supplementary material for: Potential normal tissue sparing in vulvar cancer using intensity modulated proton therapy versus volumetric modulated arc therapy
Source: Phys Imaging Radiat Oncol. 2026 May 27;39:101004. doi: 10.1016/j.phro.2026.101004 (PMC13255043; doi:10.1016/j.phro.2026.101004)
Supplement: Supplementary Data 1 — Supplementary Information including additional methodological details, analyses, and supporting results. [file mmc1.pdf]

Supplementary A

Methods and Materials

Inclusion criteria

Patient data were collected and anonymized through informed consent procedures. Inclusion criteria were histologically confirmed locally advanced or recurrent disease, treatment with primary or adjuvant curative (chemo)radiotherapy, inclusion of a tumor boost, regional, and bilateral inguinal lymph nodes in the clinical target volume (CTV), and sufficient quality of computed tomography (CT) imaging for treatment planning.

Target and normal tissue delineation

The GTV was based on gynecological examination and MRI/PET tumor extension. Vulvar elective CTV included the entire vulva and the mons pubis. The lymph node elective CTV was comprised of the inguinofemoral region containing both superficial and deep inguinal lymph nodes, including lymphoceles and visible lymph nodes, as well as the arteria iliaca externa and interna, and fossa obturatoria, according to the RTOG atlas [1]. Median target volume dimensions for the cohort are listed in Supplementary Table S3.A.2.

Bladder, femoral heads and anorectum were delineated. The bowel bag was automatically delineated using the MIM Contour Protégé AI+ model [2] and manually adjusted when necessary. Bone marrow, including iliac crests, lumbosacral spine bone marrow (LSSBM), lower pelvis bone marrow (LPBM), and whole pelvic bone marrow (WPBM), were contoured according to standardized guidelines, described in [3, 4]. The skin was defined from the surface of the patient as a shell with 3 mm thickness.

Normal tissue complication probability (NTCP)

NTCP models for vulvar cancer are lacking. For an exploratory analysis, eight NTCP models of different side effect endpoints across relevant organs of interest (OOI) from different disease sites were applied to the plans to estimate the probability of radiation-induced adverse events. Endpoints were: acute urinary urgency grade ≥ 2 (bladder) [5], diarrhea grade ≥ 1 (bowel bag) [6], acute grade ≥ 2 GI side effects (bowel bag) [7], late persistent GI side effects (bowel bag) [8], necrosis (femoral heads) [9], radiation induced urgency syndrome (rectum) [10], hematologic side effects grade ≥ 3 (WPBM) [11] and grade 3 radiation dermatitis (skin) [12]. Input parameters were either the equivalent uniform dose (EUD) or other dose–volume histogram (DVH) metrics, except for the skin model, which used relative dose–surface histograms (DSHs). Absolute DSHs were calculated according to the method of Palma et al. [13] and normalized using the Body Surface Area (BSA) index [14] as a scaling factor to account for differing patient sizes. The relative skin surface receiving more than 20 Gy(RBE) ($S_{20Gy(RBE)}$) was used as a predictor [12]. Although dose calculation uncertainties are relatively large in the first few millimeters from the body surface, Mori et al. [15] demonstrated that these uncertainties have negligible influence on logistic regression coefficients, supporting the use of this model. All NTCP functions and their variables are summarized in Supplementary S3.C.

Dose-surface skin maps

To visualize the spatial distribution of the dose to the skin, 3D dose-surface maps were generated for the VMAT and IMPT plans using an in-house Python script. The patient's contour was mapped to a 3D surface mesh and dose values were sampled onto the mesh using tri-linear interpolation of the dose grid. Colors were assigned based on the local dose value using a custom colormap. Both low (5–25 Gy(RBE)) and high-dose (≥ 25 Gy(RBE)) surface maps were created.

References

[1] D. K. Gaffney et al., Consensus recommendations for radiation therapy contouring and treatment of vulvar carcinoma, *Int J Radiat Oncol Biol Phys* 95(4) (2016) 1191–1200.

[2] MIM Software Inc., Contour Protégé AI+™ [Computer software], https://www.mimsoftware.com/ (2024).

[3] L. K. Mell et al., Dosimetric predictors of acute hematologic toxicity in cervical cancer patients treated with concurrent cisplatin and IMRT, *Int J Radiat Oncol Biol Phys* 66(5) (2006) 1356–1365.

[4] J. Huang et al., Pelvic bone marrow sparing IMRT reduces the incidence of hematologic toxicity in cervical cancer patients, (2020).

[5] P. Mavroidis et al., Fitting NTCP models to bladder doses and acute urinary symptoms during post-prostatectomy radiotherapy, *Radiation Oncology* 13(1) (2018).

[6] G. Reinartz et al., Biophysical analysis of acute and late toxicity of radiotherapy in gastric marginal zone lymphoma, *Cancers* 13(6) (2021) 1–13.

[7] D. R. Simpson et al., Normal tissue complication probability analysis of acute GI toxicity in cervical cancer patients undergoing IMRT, *Int J Radiat Oncol Biol Phys* 83(1) (2012).

[8] A. Corbeau et al., NTCP models for GI toxicity after adjuvant (chemo)radiotherapy for cervical cancer in the PARCER trial, *ESTRO 2025*, pp. 810–811.

[9] C. Burman et al., Fitting of Normal Tissue Tolerance Data to an Analytic Function, *Radiat Oncol Biol Phys* 21 (1991) 123–135.

[10] E. Alevronta et al., Dose-response relationships of the sigmoid for urgency syndrome after gynaecological radiotherapy, *Acta Oncologica* 57(10) (2018) 1352–1358.

[11] J. G. Bazan et al., Impact of chemotherapy on NTCP models of acute hematologic toxicity in patients receiving pelvic IMRT, *Int J Radiat Oncol Biol Phys* 87(5) (2013) 983–991.

[12] G. Palma et al., NTCP Models for Severe Radiation Induced Dermatitis After IMRT or Proton Therapy, *Front Oncol* 10 (2020).

[13] G. Palma, L. Cella, A new formalism of Dose Surface Histograms for robust modelling of skin toxicity, *Physica Medica* 59 (2019) 75–78.

[14] D. Du Bois, E. F. Du Bois, A formula to estimate the approximate surface area if height and weight be known, *Jour Biol Chem* XVII (1916) 863–971.

[15] M. Mori et al., Skin DVHs predict cutaneous toxicity in Head and Neck Cancer patients treated with Tomotherapy, *Physica Medica* 59 (2019) 133–141.

Supplementary B

Methods and Materials

Simultaneous integrated boost (SIB) vs sequential boost (SEQ)

For patients treated with a SEQ schedule, corresponding IMPT plans were created in the same way: elective and boost dose were planned separately and then summed using deformable registration to obtain the total dose distribution for the nominal and voxel-wise worst-case plans, similar to what we do in the clinic to assess plans when plan adaptation is performed. For patients treated with SIB, IMPT planning was done using a single SIB plan covering both target volumes. The effect of SIB versus SEQ on target coverage and OOI doses was also evaluated, with the results provided in Supplementary 3.D and E.

Correlation between target volumes and NTCP

The effect of tumor size on NTCP was studied by examining the correlation between NTCP and the volumes of the GTV, elective CTV, and boost CTV for all endpoints. This was done through calculating the Spearman's rank correlation coefficient ρ [1] and performing linear regression between NTCP and the log-transformed target volumes. This analysis also aimed to identify potential trends in NTCP with increasing target volume, even in cases where the correlation was weak.

Results

SIB vs SEQ

Additional results comparing SIB and SEQ are summarized in Supplementary 3.D and E, showing target coverage and doses to the OOIs based on the constraints, respectively. For target coverage, SIB plans (Supplementary Table S3.D.1) achieved slightly higher median $D_{98\%}$ values with IMPT compared to VMAT, whereas SEQ plans (Supplementary Table S3.D.2) showed similar $D_{98\%}$ values. $D_{2\%}$ values for the boost target were comparable across SIB and SEQ.

For OOIs (SIB: Supplementary Table S3.E.2, SEQ: Supplementary Table S3.E.3), both SIB and SEQ achieved significant dose reductions for IMPT compared to VMAT, but both the magnitude and the number of constraints with statistically significant differences were generally greater in SIB. For example, in SIB, reductions in bladder $D_{35\%}$ and $D_{50\%}$ with IMPT were larger than SEQ (median decreases of ~12–18 Gy(RBE) vs ~10–15 Gy(RBE)). Bladder $V_{45Gy(RBE)}$ and bowel bag $V_{45Gy(RBE)}$ reached statistical significance only in SIB. Femoral head median doses were consistently lower in SIB, and LPBM $V_{5Gy(RBE)}$ reductions were greater in SIB (median decrease ~50% vs ~30–35%).

Correlation between target volumes and NTCP

Figure S1 shows the plot between NTCP and GTV, boost CTV and elective CTV on a log10 scale for two endpoints: late persistent gastrointestinal (GI) side effects (bowel bag) and grade 3 radiation dermatitis (skin). For both endpoints, correlations between volume and NTCP were weak (Spearman's ρ = 0.18–0.52, R^2 < 0.3). Nevertheless, a trend toward higher NTCP with increasing target volumes was observed. This increase was similar or smaller for IMPT than for VMAT. Data for the remaining six endpoints are shown in Supplementary S3.G, showing the same trend observed in bowel bag and skin for all endpoints.

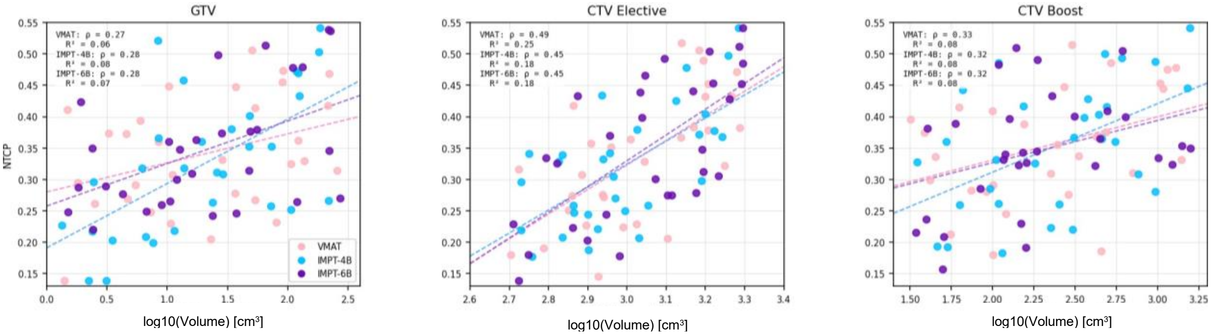

(a) Late persistent GI side effects (Bowel Bag)

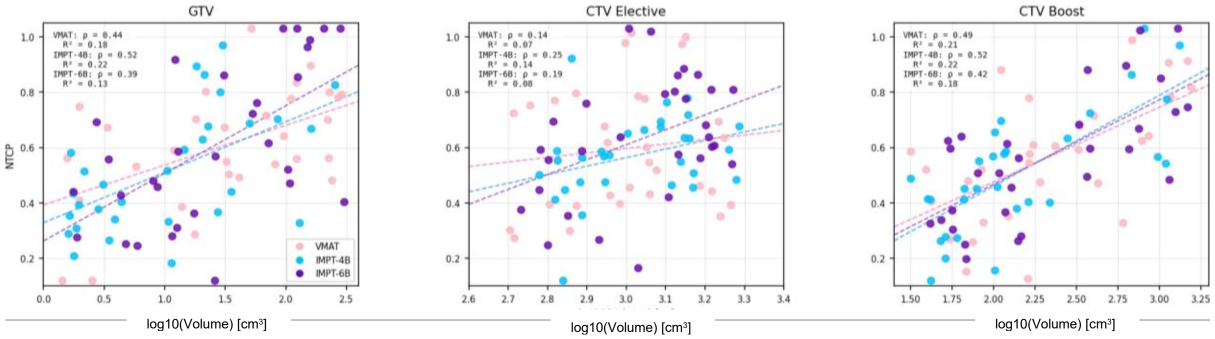

(b) Grade 3 radiation dermatitis (Skin)

Figure S1: NTCP versus the target volumes GTV, elective CTV and boost CTV in volume (cm³) on a log10 scale, for the treatment modalities VMAT, IMPT-4B and IMPT-6B. *Linear regression lines are shown as dashed lines. Spearman's rank correlation coefficient (ρ) and the coefficient of determination (R^2) are reported in the top left corner. Two endpoints are shown here: late persistent gastrointestinal side effects (a) and grade 3 radiation dermatitis (b). The remaining six endpoints are shown in Supplementary S3.G.*

Discussion

SIB vs SEQ

While our primary focus was the comparison between VMAT and IMPT, we also compared the difference between SIB and SEQ delivery approaches. Both SIB and SEQ achieved robust target coverage and substantial OOIs sparing with IMPT compared to VMAT. However, SIB generally produced steeper dose gradients and larger reductions in several OOIs constraints. These differences are likely related to the direct optimization of the target coverage for both boost and elective regions in SIB planning, which allows better control over coverage and dose fall-off than summing the dose afterwards in SEQ. SIB fractionation may therefore be the preferable option for IMPT planning in vulvar cancer, and these findings are in line with other studies where SIB achieved superior or similar target coverage and OOIs sparing compared with SEQ [2, 3]. Nevertheless, IMPT maintained its relative advantage over VMAT regardless of the boost delivery method.

Correlation between target volumes and NTCP

An additional NTCP correlation analysis was performed to investigate the relationship between three target volumes (GTV, elective CTV, boost CTV) and NTCP. A slight positive correlation was observed between elective CTV volume and late persistent GI side effects risk, and between GTV/boost CTV volume and grade 3 radiation dermatitis risk. For the other endpoints, correlations were weak, as observed in Supplementary S3.G. Despite these correlations, the low R^2 values indicate that target volume explains only a small part of the variance in NTCP, suggesting that other clinical or anatomical factors likely play a larger role. When examining trends, NTCP generally increases with larger target volumes, but the magnitude of this increase was similar or lower for IMPT compared to VMAT. This indicates that IMPT plans may be less sensitive to target volume changes and could therefore maintain a dosimetric advantage even in patients with larger tumors.

References

[1] C. Spearman, The Proof and Measurement of Association between Two Things, *The American Journal of Psychology* 15(1) (1904) 72.

[2] X. R. Zhu et al., A single-field integrated boost treatment planning technique for spot scanning proton therapy, *Radiation Oncology* 9(1) (2014).

[3] P. Franco et al., Comparing simultaneous integrated boost vs sequential boost in anal cancer patients: results of a retrospective observational study, *Radiation Oncology* 13(1) (2018) 172.

Supplementary 3.A. Patient and Target Volume Characteristics

Table S3.A.1: Patient characteristics.

Parameter	Median (range) / n (%)
Age at RT (years)	64.5 (45–84)
Weight (kg)	73.5 (54.7–132.0)
Height (cm)	166 (148–183)
BSA (m²)	1.85 (1.49–2.41)
**Smoking status**	**n (%)**
Never	14 (47%)
Prior	7 (23%)
Current	9 (30%)
**HPV status**	**n (%)**
Positive	10 (33%)
Negative	16 (53%)
Unknown	4 (13%)
**Radiotherapy**	**n (%)**
Primary, SIB	10 (33%)
Primary, SEQ	16 (53%)
Adjuvant, SIB	4 (13%)
**Chemotherapy**	**n (%)**
Mitomycin-c/capecitabine	11 (37%)
5-fluorouracil/mitomycin-c	17 (57%)
Carboplatin	1 (1%)
None	1 (1%)
**Tumor stage**	**n (%)**
T1	13 (43%)
T2	13 (43%)
T3	1 (3%)
T4	2 (7%)
**Nodal stage**	**n (%)**
N0	15 (50%)
N1	6 (20%)
N2	6 (20%)
N3	3 (10%)
**Distant metastasis**	**n (%)**
M0	29 (97%)
M1	1 (3%)

Table S3.A.2: Target volume dimensions. *The right column indicates for how many patients this structure was delineated.*

Structure	Median volume (cm³)	Range (cm³)	n (%)
Vulvar GTV	38.0	4.7–416.3	25 (83.3%)
Nodal GTV	11.1	0.9–69.2	13 (43.3%)
Vulvar Elective CTV	508.9	129.4–1614.8	30 (100%)
Nodal Elective CTV	689.5	267.9–916.3	30 (100%)
Vulvar Boost CTV	157.5	35.1–1223.6	26 (86.7%)
Nodal Boost CTV	45.3	1.5–493.4	18 (60%)

Supplementary 3.B. Target and Organs of Interest (OOI) Constraints

Table S3.B.1: Target and organs of interest constraints applied for evaluation of the treatment plans. *Dose at volume constraints, like $D_{5\%}$ < 50 Gy(RBE), mean that D% of the OOI/target should receive less/more than the stated dose or stated percentage of prescribed dose. Volume at dose constraints, like $V_{45Gy(RBE)}$ < 60%, mean that the volume that receives VGy(RBE) dose should receive less than the stated volume or stated percentage of the OOI.*

Target constraints

Target	Constraint 1	Constraint 2
Boost [1]	$D_{98\%} \geq 95\%$	$D_{2\%} \leq 107\%$
Elective [1]	$D_{98\%} \geq 95\%$	–

OOI constraints

OOI	Constraint 1	Constraint 2	Constraint 3	Constraint 4
Bladder [2,3]	$D_{5\%}$ < 50 Gy(RBE)	$D_{35\%}$ < 40 Gy(RBE)	$D_{50\%}$ < 35 Gy(RBE)	$V_{45Gy(RBE)}$ < 50%
Bowel Bag [2]	$V_{45Gy(RBE)}$ < 20 cm^3	$V_{35Gy(RBE)}$ < 150 cm^3	$V_{30Gy(RBE)}$ < 200 cm^3	–
Anorectum [4,3]	$V_{45Gy(RBE)}$ < 60%	$V_{40Gy(RBE)}$ < 80%	–	–

Bone constraints

Bone OOI	Constraint 1	Constraint 2	Constraint 3
Femoral heads [2]	$D_{5\%}$ < 44 Gy(RBE)	$D_{35\%}$ < 40 Gy(RBE)	$D_{50\%}$ < 30 Gy(RBE)
Iliac crests [2]	$D_{5\%}$ < 50 Gy(RBE)	$D_{35\%}$ < 40 Gy(RBE)	$D_{50\%}$ < 30 Gy(RBE)
WP Bone marrow [5]	$V_{40Gy(RBE)}$ < 28%	–	–
LP Bone marrow [5]	$V_{5Gy(RBE)}$ < 95%	$V_{20Gy(RBE)}$ < 45%	–
LSS Bone marrow [6]	D_{mean} < 39 Gy(RBE)	$V_{10Gy(RBE)}$ < 87%	–

WP = whole pelvis, LP = lower pelvis, LSS = lumbosacral spine

Supplementary 3.C. Normal Tissue Complication Probability (NTCP) Models

To estimate probability of normal tissue complications, three types of NTCP models were used in this study: the Lyman-Kutcher-Burman model, the logistic-type function model, and the multivariate regression model.

Lyman-Kutcher-Burman (LKB) Model

NTCP in the LKB model [9, 15, 16] is calculated by:

$$NTCP = (1/\sqrt{2\pi}) \int_{-\infty}^{t} e^{-x^2/2} dx$$

$$\text{with: } t = (EUD - TD50) / (m \cdot TD50)$$

where TD50 is the dose at which 50% of the volume experiences complications, m is a tissue-specific slope parameter, and EUD represents the equivalent uniform dose. The EUD converts the dose-volume-histogram (DVH) to a homogeneous dose and is calculated as:

$$EUD = (\Sigma_i v_i \cdot D_i^{1/n})^n$$

where D_i is the dose in subvolume v_i and n is a tissue-specific parameter that accounts for the volume effect.

Logistic-Type Function Model

The logistic-type function model chooses NTCP as a sigmoid shape function. Simpson et al. [12] define their model for gastrointestinal side effects as follows:

$$NTCP = \exp(4\gamma(V45/V50 - 1)) / [1 + \exp(4\gamma(V45/V50 - 1))]$$

where V45 is the volume of bowel receiving ≥ 45 Gy, V50 is the volume at which 50% complication probability occurs and γ is the normalized slope of the volume-response curve. For this study, the model was applied to the bowel bag instead of the bowel.

Multivariate Logistic Regression Modelling

While most NTCP models use one dose-variable, (g)EUD, the spatial distribution of dose may carry important information as well. Next to this, treatment and patient characteristics may also affect normal tissue complications. Multivariate NTCP models [14] aim to predict toxicities by including non-dose related factors. NTCP is estimated through multivariate logistic regression analysis using the following general equation:

$$NTCP = 1 / (1 + e^{-S})$$

where S is an equation in the form $S = \beta0 + (x1\beta1, ..., xn\beta n)$, that includes the parameters of the model. The NTCP models and their parameters that are used in this study are listed in Table S3.C.1.

Table S3.C.1: Used NTCP models

Lyman-Kutcher-Burman Models

Model	OOI	Endpoint	n	m	TD50 (Gy)
Mavroidis 2018 [7]	Bladder	Acute urinary urgency grade ≥ 2	1	0.5	64.2
Reinartz 2021 [8]	Bowel Bag	Diarrhea grade ≥ 1	0.15	0.79	55
Burman 1991 [9]	Femoral heads	Necrosis	0.25	0.12	65
Alevronta 2018 [10]	Anorectum	Radiation induced urgency syndrome	$9x10^6$	0.47	51.5
Bazan 2013 [11]	WPBM	Hematologic side effects grade ≥ 3	1	0.27	35

Logistic-Type Function Models

Model	OOI	Endpoint	γ	V50 (cm^3)
Simpson 2012 [12]	Bowel Bag	Acute grade ≥ 2 GI side effects	0.31	161

Logistic Regression Models

Model	OOI	Endpoint	Model variable(s)	β0	βn
Corbeau 2025 [13]	Bowel Bag	Late persistent GI side effects	$V_{30Gy(RBE)}$ (cm3), $V_{40Gy(RBE)}$ (cm3)	−1.874	$9.88x10^{-4}$, $9.65x10^{-4}$
Palma 2020 [14]	Skin	Grade 3 radiation dermatitis	$S_{20Gy(RBE)}$ (%)	−6.34	3.45

GI = gastrointestinal, WPBM = Whole pelvis bone marrow

Supplementary 3.D. Comparison of Target Coverage Between SIB and SEQ Planning Methods

Table S3.D.1: Comparison of target coverage metrics between VMAT, IMPT-4B and IMPT-6B for the patients planned with a simultaneous integrated boost (SIB) fractionation schedule. *VMAT treatment plans were evaluated on the PTV of the nominal plans, IMPT plans on the CTV on the voxel-wise minimum and maximum dose distributions ($Vox_{min/max}$). Values are median (%) [range], expressed as percentage of the prescribed dose for that target. The p-values compare VMAT with IMPT-4B, VMAT with IMPT-6B and IMPT-4B with IMPT-6B, respectively. Significant p-values are indicated in bold.*

Target	Constraint	VMAT (PTV nominal) Median [range] (%)	IMPT 4 Beams (CTV Voxmin/max) Median [range] (%)	IMPT 6 Beams (CTV Voxmin/max) Median [range] (%)	p-value (VMAT vs IMPT-4B)	p-value (VMAT vs IMPT-6B)	p-value (IMPT-4B vs IMPT-6B)
Boost	$D_{98\%} \geq 95\%$	95.2 [94.6–98.3]	96.4 [95.0–98.9]	96.6 [95.3–97.6]	**0.017**	**0.005**	**0.020**
	$D_{2\%} \leq 107\%$	104.1 [103.0–104.8]	105.6 [104.3–106.3]	105.3 [104.2–105.9]	**< 0.001**	**< 0.001**	**0.017**
Elective	$D_{98\%} \geq 95\%$	95.7 [95.1–96.8]	97.1 [96.6–98.8]	97.2 [96.9–97.5]	**< 0.001**	**< 0.001**	0.091

Table S3.D.2: Comparison of target coverage metrics between VMAT, IMPT-4B and IMPT-6B for the patients planned with a sequential boost (SEQ) fractionation schedule. *VMAT treatment plans were evaluated on the PTV of the nominal plans, IMPT plans on the CTV on the voxel-wise minimum and maximum dose distributions ($Vox_{min/max}$). Values are median (%) [range], expressed as percentage of the prescribed dose for that target. The p-values compare VMAT with IMPT-4B, VMAT with IMPT-6B and IMPT-4B with IMPT-6B, respectively. Significant p-values are indicated in bold.*

Target	Constraint	VMAT (PTV nominal) Median [range] (%)	IMPT 4 Beams (CTV Voxmin/max) Median [range] (%)	IMPT 6 Beams (CTV Voxmin/max) Median [range] (%)	p-value (VMAT vs IMPT-4B)	p-value (VMAT vs IMPT-6B)	p-value (IMPT-4B vs IMPT-6B)
Boost	$D_{98\%} \geq 95\%$	97.4 [95.4–98.6]	97.5 [97.1–97.9]	97.7 [97.3–98.1]	0.464	0.074	**< 0.001**
	$D_{2\%} \leq 107\%$	102.3 [101.7–103.6]	105.7 [104.0–107.7]	105.3 [100.3–106.7]	**< 0.001**	**< 0.001**	**0.025**
Elective	$D_{98\%} \geq 95\%$	98.6 [97.0–104.6]	97.8 [96.0–98.9]	98.2 [96.2–100.3]	**< 0.001**	**0.013**	**< 0.001**

Supplementary 3.E. Organs of Interest (OOI) Constraint Evaluation

Supplementary 3.E.1. All Patients

Table S3.E.1: Evaluation of organs of interest constraints for all patients. *Values are presented as median [range] in Gy(RBE) for dose-based constraints, and % or cm^3 for volume-based constraints. The p-values compare VMAT with IMPT-4B, VMAT with IMPT-6B and IMPT-4B with IMPT-6B, respectively. Significant p-values are indicated in bold.*

OOI	Constraint	VMAT Median [range]	IMPT-4B Median [range]	IMPT-6B Median [range]	p-value (VMAT vs IMPT-4B)	p-value (VMAT vs IMPT-6B)	p-value (IMPT-4B vs IMPT-6B)
Bladder	$D_{5\%}$ < 50 Gy(RBE)	45.4 [30.3–56.9]	44.8 [19.6–53.3]	45.0 [18.5–52.8]	**0.031**	**0.014**	**0.001**
	$D_{35\%}$ < 40 Gy(RBE)	33.6 [17.9–48.1]	23.1 [4.8–39.9]	22.8 [4.9–39.9]	**< 0.001**	**< 0.001**	0.339
	$D_{50\%}$ < 35 Gy(RBE)	31.0 [15.3–47.3]	14.9 [3.6–35.2]	15.8 [3.3–35.5]	**< 0.001**	**< 0.001**	**0.005**
	$V_{45Gy(RBE)}$ < 50%	5.8 [0.0–88.1]	4.8 [0.0–18.0]	5.0 [0.0–17.8]	**0.011**	**0.003**	**< 0.001**
Bowel Bag	$V_{30Gy(RBE)}$ < 200 cm^3	251 [16.7–299]	181 [6.0–239]	182 [67.3–236]	**< 0.001**	**< 0.001**	0.129
	$V_{35Gy(RBE)}$ < 150 cm^3	171 [15.0–203]	138 [8.6–168]	138 [9.4–168]	**< 0.001**	**< 0.001**	0.416
	$V_{45Gy(RBE)}$ < 20 cm^3	20.6 [14.0–25.5]	20.1 [14.9–24.5]	20.0 [14.8–24.4]	**0.014**	**0.001**	**< 0.001**
Anorectum	$V_{45Gy(RBE)}$ < 60%	9.5 [0.0–54.0]	7.6 [0.0–32.0]	6.6 [0.0–30.8]	**0.007**	**< 0.001**	**< 0.001**
	$V_{40Gy(RBE)}$ < 80%	16.8 [0.2–78.6]	12.5 [0.0–41.3]	12.4 [0.0–40.0]	**0.027**	**< 0.001**	**< 0.001**
Femoral heads	$D_{5\%}$ < 44 Gy(RBE)	37.5 [31.4–48.8]	15.5 [6.8–33.7]	15.7 [8.1–35.1]	**< 0.001**	**< 0.001**	**0.011**
	$D_{35\%}$ < 40 Gy(RBE)	29.5 [21.8–35.3]	2.9 [1.4–6.4]	4.8 [3.0–7.7]	**< 0.001**	**< 0.001**	**< 0.001**
	$D_{50\%}$ < 30 Gy(RBE)	26.8 [17.7–32.2]	1.9 [0.4–4.3]	3.8 [1.7–6.3]	**< 0.001**	**< 0.001**	**< 0.001**
Iliac crests	$D_{5\%}$ < 50 Gy(RBE)	42.7 [34.1–53.2]	41.4 [30.0–50.2]	41.5 [31.2–50.6]	**< 0.001**	**< 0.001**	0.14
	$D_{35\%}$ < 40 Gy(RBE)	25.9 [6.9–36.0]	17.0 [1.3–24.5]	14.7 [2.1–23.3]	**< 0.001**	**< 0.001**	**< 0.001**
	$D_{50\%}$ < 30 Gy(RBE)	20.5 [2.5–30.3]	8.9 [0.3–19.2]	9.7 [0.3–15.0]	**< 0.001**	**< 0.001**	0.452
WPBM	$V_{40Gy(RBE)}$ < 28%	13.7 [3.7–27.1]	8.4 [3.2–14.8]	8.0 [3.3–14.5]	**< 0.001**	**< 0.001**	**0.025**
LPBM	$V_{5Gy(RBE)}$ < 95%	100 [100–100]	52.8 [31.7–76.5]	62.9 [39.1–97.8]	**< 0.001**	**< 0.001**	**0.005**
	$V_{20Gy(RBE)}$ < 45%	94.8 [66.6–100]	27.2 [12.5–39.5]	27.6 [12.9–39.1]	**< 0.001**	**< 0.001**	**< 0.001**
LSSBM	$V_{10Gy(RBE)}$ < 87%	71.7 [7.5–100]	62.8 [0.7–99.8]	60.4 [0.2–99.5]	**< 0.001**	**< 0.001**	**< 0.001**
	D_{mean} < 39 Gy(RBE)	19.6 [2.8–34.2]	16.4 [0.7–34.3]	14.3 [0.5–30.6]	**< 0.001**	**< 0.001**	**< 0.001**

WPBM = whole pelvic bone marrow, LPBM = lower pelvic bone marrow, LSSBM = lumbosacral spine bone marrow

Supplementary 3.E.2. SIB Patients

Table S3.E.2: Evaluation of organs of interest constraints for patients with a SIB fractionation schedule. *Values are presented as median [range] in Gy(RBE) for dose-based constraints, and % or cm^3 for volume-based constraints. The p-values compare VMAT with IMPT-4B, VMAT with IMPT-6B and IMPT-4B with IMPT-6B, respectively. Significant p-values are indicated in bold.*

OOI	Constraint	VMAT Median [range]	IMPT-4B Median [range]	IMPT-6B Median [range]	p-value (VMAT vs IMPT-4B)	p-value (VMAT vs IMPT-6B)	p-value (IMPT-4B vs IMPT-6B)
Bladder	$D_{5\%}$ < 50 Gy(RBE)	45.4 [41.0–56.9]	45.6 [38.2–49.9]	45.7 [37.5–49.1]	0.463	0.358	**0.005**
	$D_{35\%}$ < 40 Gy(RBE)	33.3 [23.3–48.1]	23.9 [13.1–39.9]	23.5 [13.1–39.9]	**< 0.001**	**< 0.001**	0.67
	$D_{50\%}$ < 35 Gy(RBE)	30.8 [20.0–47.3]	15.4 [8.5–35.2]	16.5 [8.7–35.5]	**< 0.001**	**< 0.001**	0.078
	$V_{45Gy(RBE)}$ < 50%	5.8 [1.8–88.1]	5.8 [1.3–13.7]	5.9 [1.1–13.0]	0.296	0.104	**0.004**
Bowel Bag	$V_{30Gy(RBE)}$ < 200 cm^3	258 [191–290]	182 [76.7–239]	182 [81.3–236]	**< 0.001**	**< 0.001**	0.194
	$V_{35Gy(RBE)}$ < 150 cm^3	175 [139–191]	140 [77.1–168]	139 [79.2–165]	**< 0.001**	**< 0.001**	0.583
	$V_{45Gy(RBE)}$ < 20 cm^3	21.0 [19.7–21.6]	20.8 [19.2–21.7]	20.6 [19.0–21.5]	0.217	0.078	**< 0.001**
Anorectum	$V_{45Gy(RBE)}$ < 60%	6.2 [1.8–28.1]	5.3 [1.8–13.4]	4.7 [1.4–12.4]	**0.035**	**0.013**	**0.003**
	$V_{40Gy(RBE)}$ < 80%	14.3 [6.4–66.2]	9.9 [3.6–21.8]	9.6 [3.6–18.8]	**< 0.001**	**< 0.001**	0.358
Femoral heads	$D_{5\%}$ < 44 Gy(RBE)	35.9 [31.4–48.8]	14.4 [6.8–33.7]	14.4 [8.1–35.1]	**< 0.001**	**< 0.001**	**0.025**
	$D_{35\%}$ < 40 Gy(RBE)	27.5 [23.3–34.3]	2.4 [1.4–4.6]	4.1 [3.0–6.1]	**< 0.001**	**< 0.001**	**< 0.001**

OOI	Constraint	VMAT Median [range]	IMPT-4B Median [range]	IMPT-6B Median [range]	p-value (VMAT vs IMPT-4B)	p-value (VMAT vs IMPT-6B)	p-value (IMPT-4B vs IMPT-6B)
	$D_{50\%}$ < 30 Gy(RBE)	25.2 [20.4–30.7]	1.6 [0.7–2.7]	3.0 [1.7–4.7]	**< 0.001**	**< 0.001**	**< 0.001**
Iliac crests	$D_{5\%}$ < 50 Gy(RBE)	43.2 [38.3–47.1]	42.2 [30.7–44.2]	42.2 [31.2–44.3]	**< 0.001**	**< 0.001**	0.67
	$D_{35\%}$ < 40 Gy(RBE)	26.7 [22.7–32.9]	16.8 [7.4–22.0]	14.9 [9.8–18.7]	**< 0.001**	**< 0.001**	**0.011**
	$D_{50\%}$ < 30 Gy(RBE)	21.9 [10.1–26.3]	9.3 [1.4–15.7]	10.2 [4.9–14.1]	**< 0.001**	**< 0.001**	0.153
WPBM	$V_{40Gy(RBE)}$ < 28%	11.9 [6.1–27.1]	8.4 [3.2–13.7]	7.6 [3.3–13.5]	**< 0.001**	**< 0.001**	**0.004**
LPBM	$V_{5Gy(RBE)}$ < 95%	100.0 [100.0–100.0]	48.0 [31.7–62.8]	51.0 [39.1–75.5]	**< 0.001**	**< 0.001**	**0.035**
	$V_{20Gy(RBE)}$ < 45%	91.7 [66.6–100.0]	25.4 [16.3–33.8]	25.1 [16.6–34.1]	**< 0.001**	**< 0.001**	**< 0.001**
LSSBM	$V_{10Gy(RBE)}$ < 87%	69.1 [46.5–100.0]	59.5 [26.2–96.5]	55.2 [23.5–96.2]	**< 0.001**	**< 0.001**	**< 0.001**
	D_{mean} < 39 Gy(RBE)	20.5 [11.3–34.2]	16.1 [7.7–32.0]	13.3 [6.8–28.1]	**< 0.001**	**< 0.001**	**< 0.001**

WPBM = whole pelvic bone marrow, LPBM = lower pelvic bone marrow, LSSBM = lumbosacral spine bone marrow

Supplementary 3.E.3. SEQ Patients

Table S3.E.3: Evaluation of organs of interest constraints for patients with a SEQ fractionation schedule. *Values are presented as median [range] in Gy for dose-based constraints, and % or cm^3 for volume-based constraints. Significant p-values are indicated in bold.*

OOI	Constraint	VMAT Median [range]	IMPT-4B Median [range]	IMPT-6B Median [range]	p-value (VMAT vs IMPT-4B)	p-value (VMAT vs IMPT-6B)	p-value (IMPT-4B vs IMPT-6B)
Bladder	$D_{5\%}$ < 50 Gy(RBE)	45.7 [30.3–56.4]	44.2 [19.6–53.3]	44.2 [18.5–52.8]	0.051	**0.029**	0.083
	$D_{35\%}$ < 40 Gy(RBE)	33.8 [17.9–44.2]	20.2 [4.8–38.8]	21.6 [4.9–38.4]	**< 0.001**	**< 0.001**	0.375
	$D_{50\%}$ < 35 Gy(RBE)	31.0 [15.3–38.7]	12.7 [3.6–31.0]	15.2 [3.3–30.8]	**< 0.001**	**< 0.001**	**0.034**
	$V_{45Gy(RBE)}$ < 50%	5.7 [0.0–32.1]	3.9 [0.0–18.0]	3.9 [0.0–17.8]	**0.017**	**0.015**	**0.005**
Bowel Bag	$V_{30Gy(RBE)}$ < 200 cm^3	242 [16.7–299]	180 [6.0–235]	182 [6.7–235]	**< 0.001**	**< 0.001**	0.404
	$V_{35Gy(RBE)}$ < 150 cm^3	165 [15.0–203]	136 [8.6–167]	137 [9.4–168]	**< 0.001**	**< 0.001**	0.669
	$V_{45Gy(RBE)}$ < 20 cm^3	20.0 [14.0–25.5]	19.5 [14.9–24.5]	19.4 [14.8–24.4]	**0.039**	**0.011**	**< 0.001**
Anorectum	$V_{45Gy(RBE)}$ < 60%	14.1 [0.0–54.0]	11.3 [0.0–32.0]	10.2 [0.0–30.8]	0.055	**0.018**	**0.022**
	$V_{40Gy(RBE)}$ < 80%	23.0 [0.2–78.6]	14.5 [0.0–41.3]	13.7 [0.0–40.0]	**0.002**	**0.002**	**0.041**
Femoral heads	$D_{5\%}$ < 44 Gy(RBE)	40.7 [33.3–46.8]	20.6 [10.4–31.6]	20.7 [11.6–32.0]	**< 0.001**	**< 0.001**	0.159
	$D_{35\%}$ < 40 Gy(RBE)	30.7 [21.8–35.3]	3.9 [1.4–6.4]	6.1 [3.9–7.7]	**< 0.001**	**< 0.001**	**< 0.001**
	$D_{50\%}$ < 30 Gy(RBE)	28.1 [17.7–32.2]	2.2 [0.4–4.3]	4.6 [2.5–6.3]	**< 0.001**	**< 0.001**	**< 0.001**
Iliac crest	$D_{5\%}$ < 50 Gy(RBE)	42.2 [34.1–53.2]	40.9 [29.9–50.3]	41.3 [31.4–50.6]	**< 0.001**	**< 0.001**	0.105
	$D_{35\%}$ < 40 Gy(RBE)	25.9 [6.9–36.0]	17.0 [1.3–24.5]	14.7 [2.1–23.3]	**< 0.001**	**< 0.001**	**0.004**
	$D_{50\%}$ < 30 Gy(RBE)	19.4 [2.5–30.4]	8.6 [0.3–19.2]	9.4 [0.3–14.9]	**< 0.001**	**< 0.001**	0.821
WPBM	$V_{40Gy(RBE)}$ < 28%	15.1 [3.7–26.6]	8.5 [3.4–14.8]	8.7 [3.4–14.5]	**< 0.001**	**< 0.001**	0.706
LPBM	$V_{5Gy(RBE)}$ < 95%	100.0 [100.0–100.0]	63.0 [39.1–76.5]	70.9 [45.8–97.8]	**< 0.001**	**< 0.001**	0.065
	$V_{20Gy(RBE)}$ < 45%	96.9 [83.3–100.0]	30.9 [12.5–39.5]	30.8 [12.9–39.1]	**< 0.001**	**< 0.001**	**< 0.001**
LSSBM	$V_{10Gy(RBE)}$ < 87%	73.7 [7.5–100.0]	62.8 [0.7–99.8]	60.4 [0.2–99.5]	**< 0.001**	**< 0.001**	**< 0.001**
	D_{mean} < 39 Gy(RBE)	19.6 [2.8–33.5]	16.4 [0.7–34.3]	14.3 [0.5–30.6]	**< 0.001**	**< 0.001**	**< 0.001**

WPBM = whole pelvic bone marrow, LPBM = lower pelvic bone marrow, LSSBM = lumbosacral spine bone marrow

Supplementary 3.F. Normal Tissue Complication Probability Evaluation

Table S3.F.1: NTCP evaluation. *Values are presented as median [range] in % chance of normal tissue complications. The p-values compare VMAT with IMPT-4B, VMAT with IMPT-6B and IMPT-4B with IMPT-6B, respectively. Significant p-values are indicated in bold.*

OOI	Endpoint	VMAT Median [range] (%)	IMPT-4B Median [range] (%)	IMPT-6B Median [range] (%)	p-value (VMAT vs IMPT-4B)	p-value (VMAT vs IMPT-6B)	p-value (IMPT-4B vs IMPT-6B)
Bladder	Acute urinary urgency grade ≥ 2	16.8 [7.2–33.0]	9.4 [3.4–27.3]	9.7 [3.4–27.3]	**< 0.001**	**< 0.001**	**< 0.001**
WPBM	Hematologic side effects grade ≥ 3	19.4 [4.6–50.2]	2.3 [0.4–8.9]	2.2 [0.4–9.5]	**< 0.001**	**< 0.001**	0.253
Bowel Bag	Acute grade ≥ 2 GI side effects	63.5 [25.7–97.2]	63.8 [28.8–95.0]	62.5 [29.1–94.7]	0.626	0.440	**< 0.001**
	Late persistent GI side effects	29.2 [14.8–52.7]	23.1 [15.0–37.9]	23.0 [15.0–37.6]	**< 0.001**	**< 0.001**	**0.004**
	Diarrhea grade ≥ 1	30.6 [24.7–37.3]	30.0 [25.5–36.3]	30.1 [25.5–36.4]	**0.005**	**0.003**	0.584
Femoral heads	Necrosis	$2.1x10^{-6}$ [$4.4x10^{-8}$–$1.5x10^{-4}$]	$9.5x10^{-12}$ [$1.1x10^{-13}$–$4.7x10^{-7}$]	$1.1x10^{-11}$ [$1.3x10^{-13}$–$4.8x10^{-7}$]	**< 0.001**	**< 0.001**	0.792
Anorectum	Radiation-induced urgency syndrome	19.8 [5.6–81.4]	5.6 [2.2–81.4]	6.0 [2.2–81.4]	**< 0.001**	**< 0.001**	0.157
Skin	Grade 3 radiation dermatitis	79.6 [14.0–99.2]	76.3 [17.9–96.6]	66.9 [13.1–95.4]	0.700	**< 0.001**	**< 0.001**

GI = gastrointestinal, WPBM = Whole pelvis bone marrow

Supplementary 3.G. NTCP vs Target Volume

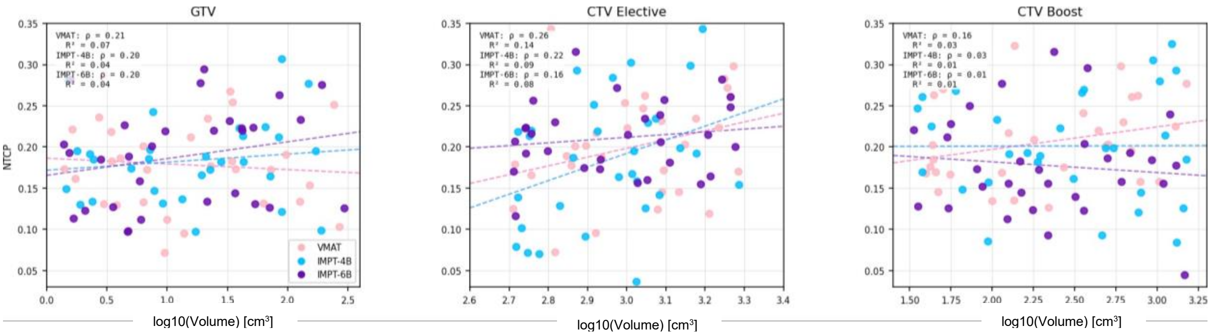

(a) Acute urinary urgency grade ≥ 2 (Bladder)

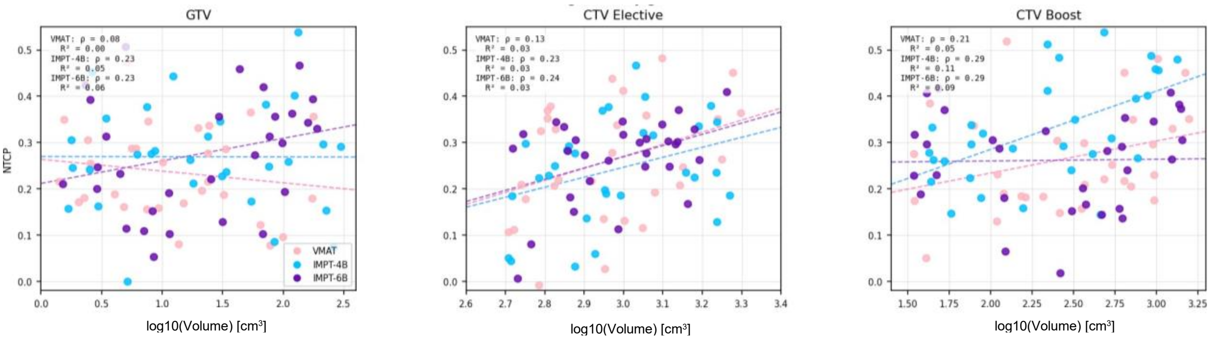

(b) Hematologic side effects grade ≥ 3 (Bone Marrow)

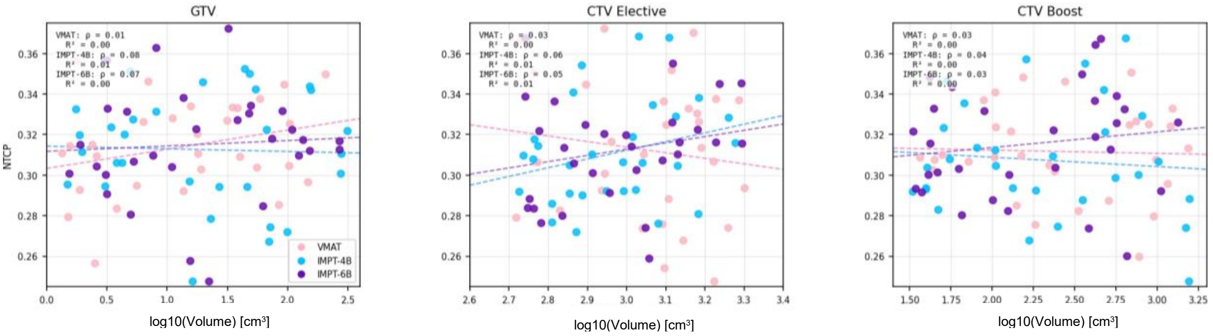

(c) Diarrhea grade ≥ 1 (Bowel Bag)

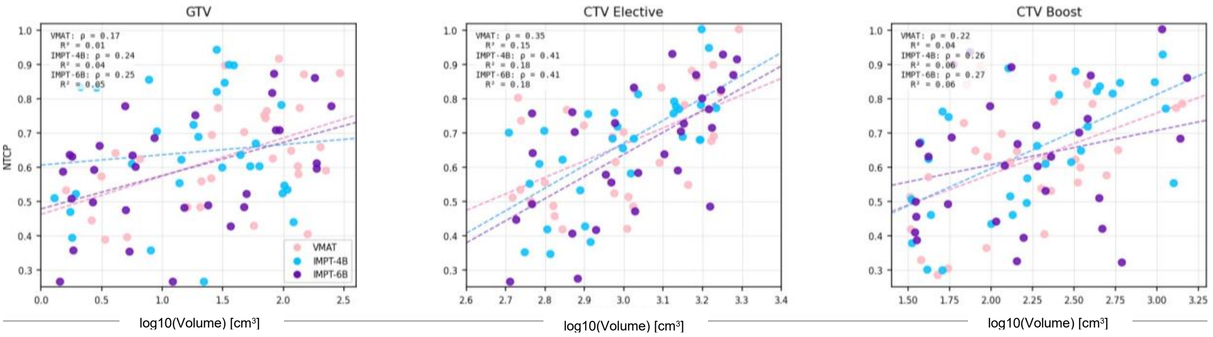

(d) Acute grade ≥ 2 gastrointestinal side effects (Bowel Bag)

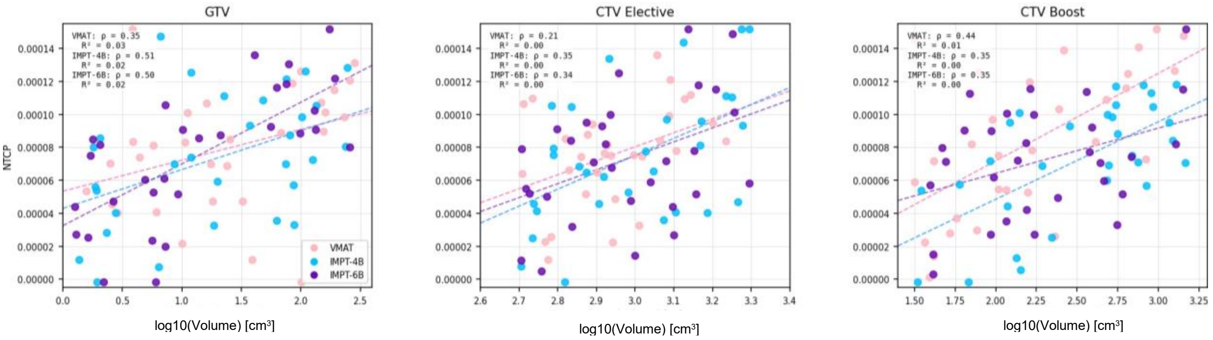

(e) Necrosis (Femoral Heads)

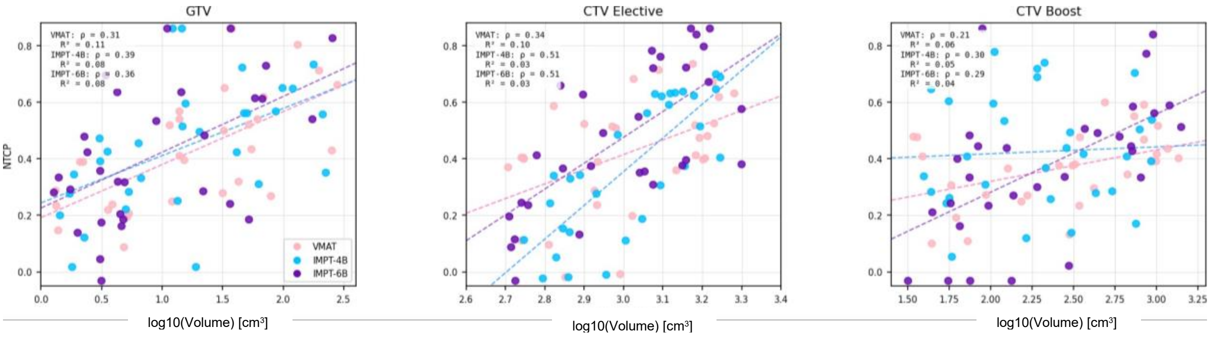

(f) Radiation induced urgency syndrome (Anorectum)

Figure S3.G.1: NTCP versus the log-10 transformed values of target volumes GTV, elective CTV and boost CTV in cm^3, for the treatment modalities VMAT, IMPT-4B and IMPT-6B. *Linear regression lines are shown as dashed lines. Spearman's rank correlation coefficient (ρ) and the coefficient of determination (R^2) are reported in the top left corner. Six endpoints (a)–(f) are shown; the remaining two endpoints are shown in Figure S1 in Supplementary 2.*

References (Supplementary 3.A–G)

[1] N. Hodapp, Der ICRU-Report 83: Verordnung, Dokumentation und Kommunikation der fluenzmodulierten Photonenstrahlentherapie (IMRT), *Strahlentherapie und Onkologie* 188(1) (2012) 97–100.

[2] L. A. Kachnic et al., RTOG 0529: A phase 2 evaluation of dose-painted IMRT in combination with 5-fluorouracil and mitomycin-C for the reduction of acute morbidity in carcinoma of the anal canal, *Int J Radiat Oncol Biol Phys* 86(1) (2013) 27–33.

[3] N. S. Horowitz et al., Phase II Trial of Cisplatin, Gemcitabine, and IMRT for Locally Advanced Vulvar Squamous Cell Carcinoma: NRG Oncology/GOG Study 279, *J Clin Oncol* 42(16) (2024) 1914–1921.

[4] A. Jhingran et al., A Phase II Study of IMRT to the Pelvis for Postoperative Patients With Endometrial Carcinoma: RTOG Trial 0418, *Int J Radiat Oncol Biol Phys* 84(1) (2012) e23–e28.

[5] J. Huang et al., Pelvic Bone Marrow Sparing IMRT Reduces the Bone Mineral Density Loss of Patients With Cervical Cancer, *Int J Radiat Oncol Biol Phys* (2024).

[6] D. Konnerth et al., Hematologic Toxicity and Bone Marrow-Sparing Strategies in Chemoradiation for Locally Advanced Cervical Cancer: A Systematic Review (2024).

[7] P. Mavroidis et al., Fitting NTCP models to bladder doses and acute urinary symptoms during post-prostatectomy radiotherapy, *Radiation Oncology* 13(1) (2018).

[8] G. Reinartz et al., Biophysical analysis of acute and late toxicity of radiotherapy in gastric marginal zone lymphoma, *Cancers* 13(6) (2021) 1–13.

[9] C. Burman et al., Fitting of Normal Tissue Tolerance Data to an Analytic Function, *Radiat Oncol Biol Phys* 21 (1991) 123–135.

[10] E. Alevronta et al., Dose-response relationships of the sigmoid for urgency syndrome after gynecological radiotherapy, *Acta Oncologica* 57(10) (2018) 1352–1358.

[11] J. G. Bazan et al., Impact of chemotherapy on NTCP models of acute hematologic toxicity in patients receiving pelvic IMRT, *Int J Radiat Oncol Biol Phys* 87(5) (2013) 983–991.

[12] D. R. Simpson et al., NTCP analysis of acute GI toxicity in cervical cancer patients undergoing IMRT and concurrent cisplatin, *Int J Radiat Oncol Biol Phys* 83(1) (2012).

[13] A. Corbeau et al., NTCP models for GI toxicity after adjuvant (chemo)radiotherapy for cervical cancer in the PARCER trial, *ESTRO 2025*, Radiotherapy and Oncology, pp. 810–811.

[14] G. Palma et al., NTCP Models for Severe Radiation Induced Dermatitis After IMRT or Proton Therapy for Thoracic Cancer Patients, *Front Oncol* 10 (2020).

[15] J. T. Lyman, Complication Probability as Assessed from Dose-Volume Histograms, *Radiation Research* 104(2) (1985) 13–19.

[16] G. J. Kutcher, C. Burman, Calculation of complication probability factors for non-uniform normal tissue irradiation: the effective volume method, *Int J Radiat Oncol Biol Phys* 16 (1989) 1623–1630.
